# Combined prognostic value of AI-derived CT-FFR and high-risk plaque characteristics in patients with newly diagnosed chronic coronary syndrome: a prospective cohort study

**DOI:** 10.3389/fcvm.2025.1674126

**Published:** 2025-12-03

**Authors:** Renjie Zhang, Wei Fu, Jianan Xu, Honghou He, Xing Guan, Yang You, Fei Lyu, Naying Jin, Xiaoyu Bai, Xiaoning Lu, Zelong Cao, Liang Zheng, Mingqi Zheng

**Affiliations:** 1 Department of Cardiology, The First Hospital of Hebei Medical University, Shijiazhuang, Hebei, China; 2 Hebei Key Laboratory of Heart and Metabolism, Shijiazhuang, Hebei, China; 3 Department of Cardiology, Handan First Hospital, Handan, Hebei, China; 4 The First Hospital of Hebei Medical University, Shijiazhuang, Hebei, China; 5 Shanghai East Hospital, Tongji University School of Medicine, Tongji University, Shanghai, China; 6 Shanghai Heart Failure Research Center, Shanghai East Hospital, Tongji University School of Medicine, Shanghai, China

**Keywords:** coronary heart disease, FFR, computed tomography (CT), high risk plaque features, prognosis

## Abstract

**Background:**

While coronary computed tomography angiography (CTA) is widely used for diagnosing chronic coronary syndrome (CCS), its potential for assessing physiological function and plaque vulnerability—through AI-derived fractional flow reserve (CT-FFR) and high-risk plaque characteristics (HRPC)—is not fully leveraged in clinical practice. The combined prognostic value of these non-invasive tools in newly diagnosed CCS patients remains underexplored.

**Objective:**

To evaluate the individual and combined prognostic value of AI-based CT-FFR and HRPC in predicting major adverse cardiovascular events (MACE) in patients with newly diagnosed CCS.

**Methods:**

In this observational cohort study, 222 inpatients newly diagnosed with CCS who were admitted for non-acute chest pain and underwent coronary CTA were included. Patients were stratified into four groups based on their CT-FFR and HRPC values. Kaplan–Meier survival curves and multivariate Cox proportional hazards models were used to assess the predictive value of CT-FFR and HRPC for MACE. Model performance was evaluated using the C-index, area under the receiver operating characteristic curve (AUC), net reclassification improvement (NRI), and integrated discrimination improvement (IDI).

**Results:**

Vessels with CT-FFR ≤0.8 had a higher prevalence and number of HRPC compared to those with CT-FFR >0.8. Over a median follow-up period of 22 months, 52 patients (23.4%) experienced MACE. Both CT-FFR ≤0.8 [hazard ratio (HR): 2.62, 95% confidence interval (CI): 1.06–6.47; *P* = 0.036] and HRPC ≥2 (HR: 2.39, 95% CI: 1.20–4.77; *P* = 0.014) independently predicted MACE. Patients with both CT-FFR ≤0.8 and HRPC ≥2 had a 6.06-fold increased risk of MACE compared to those with CT-FFR >0.8 and HRPC <2 (*P* = 0.017). Combining CT-FFR and HRPC significantly improved the predictive accuracy of risk models, reflected in increases in C-index, AUC, NRI, and IDI (*P* ≤ 0.038), providing superior predictive performance compared to using either metric alone.

**Conclusion:**

The combined use of AI-derived CT-FFR and HRPC significantly improves risk stratification in patients with newly diagnosed CCS, offering better predictive accuracy for adverse cardiovascular events. This enhanced risk assessment could enable clinicians to identify high-risk patients more effectively and tailor management strategies accordingly. Further multicenter studies are warranted to validate these findings across diverse populations.

## Introduction

Ischemic heart disease continues to be the leading cause of cardiovascular mortality worldwide, accounting for 9.24 million deaths in 2022 (1). Early identification of high-risk individuals and the development of appropriate therapeutic strategies at the time of initial diagnosis of coronary artery disease (CAD) are critical for improving patient outcomes (2–4). Coronary computed tomography angiography (CTA) is often the first-choice diagnostic modality for non-emergent CAD patients, particularly when acute myocardial infarction is not highly suspected (5–7). However, CTA's accuracy in detecting stenosis remains limited, and anatomical stenosis alone cannot fully assess cardiovascular risk because it lacks functional information (8, 9).

Invasive techniques such as fractional flow reserve (FFR) and high-risk plaque detection using intravascular ultrasound (IVUS) or optical coherence tomography (OCT) provide valuable risk assessment but are limited by their invasiveness, complexity, and high cost, especially in less developed regions (10–13). Recently, non-invasive CT-derived FFR (CT-FFR), obtained from CTA images using artificial intelligence (AI) techniques, has been shown to correlate closely with invasive FFR (14, 15). Similarly, high-risk coronary plaque characteristics (HRPC) based on CTA—such as low-density plaques, positive remodeling, napkin-ring sign, and spotty calcification—provide a safer and more cost-effective alternative to invasive techniques (16). Importantly, both CT-FFR and HRPC can be obtained in a single analysis through specialized software. Studies have confirmed that CT-FFR ≤0.80 (14, 17, 18) and HRPC ≥2 (19, 20) are key indicators of poor prognosis in patients with CAD.

Previous large-scale trials such as SCOT-HEART (21), and DISCHARGE (2, 22) primarily enrolled outpatient or lower-risk populations, establishing the prognostic value of CT-FFR and HRPC when evaluated separately. However, evidence in hospitalized patients with chronic coronary syndrome (CCS)—who generally exhibit higher event rates, greater comorbidity burdens, and more complex therapeutic decision-making—remains limited. The present study, based on a prospective inpatient registry, is among the first to comprehensively evaluate the combined prognostic utility of AI-derived CT-FFR and HRPC for predicting major adverse cardiovascular events (MACE), thereby testing their robustness and clinical translatability in a real-world high-risk setting.

However, the prognostic role of CT-FFR and HRPC in newly diagnosed CCS patients has not been fully validated. More importantly, although both metrics can now be easily obtained together, research on their combined application for risk stratification in CCS patients is still lacking. This study aims to investigate the individual and combined prognostic value of CT-FFR and HRPC in patients with newly diagnosed CCS hospitalized for non-acute chest pain, adding predictive value beyond traditional clinical risk factors and coronary stenosis severity.

## Methods

### Study design and population

**Figure 1:**
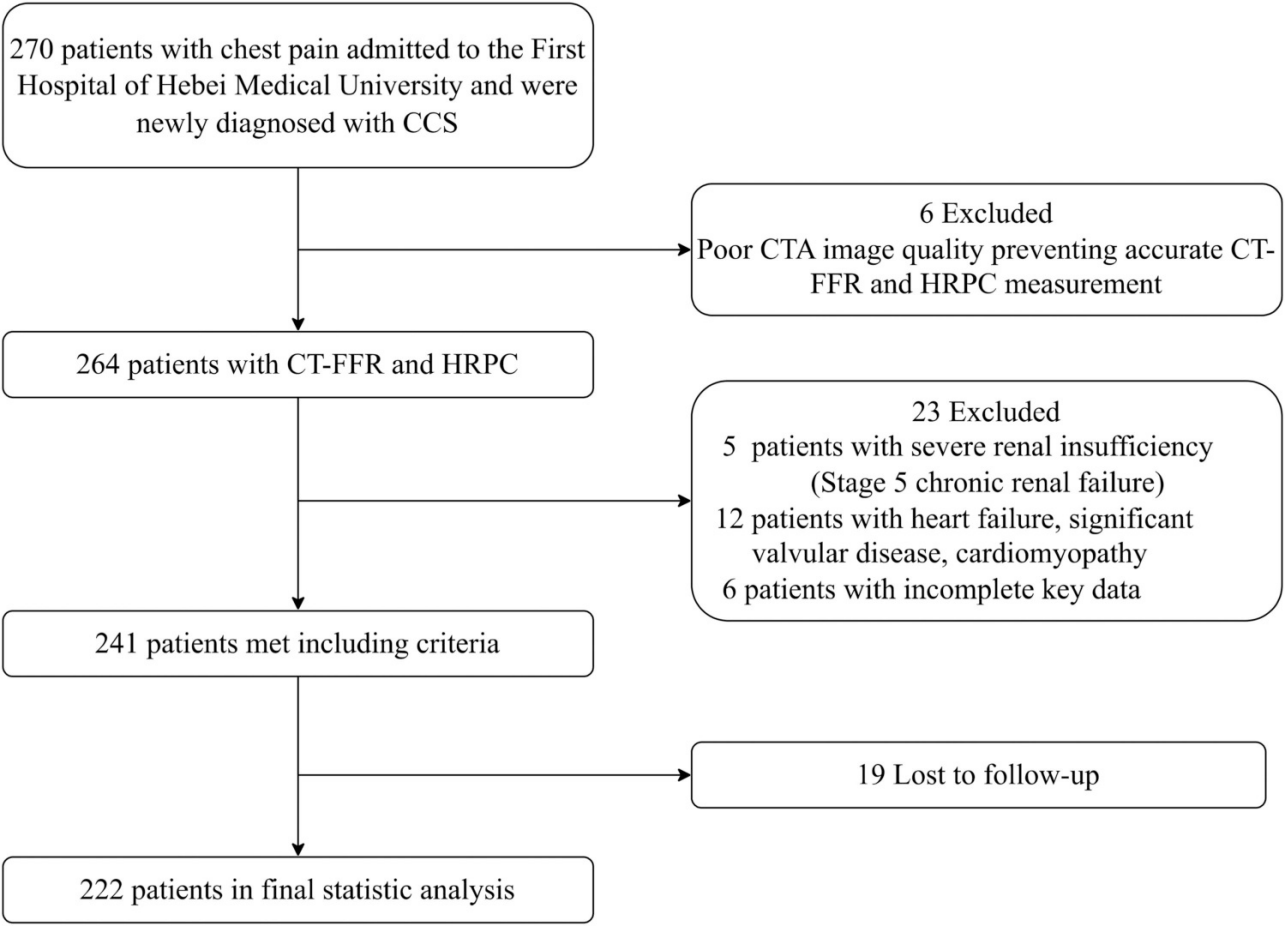
Flowchart of the study participants.

This observational cohort study enrolled patients admitted to the First Hospital of Hebei Medical University between March and September 2022 due to chest pain. All patients underwent coronary CTA and were diagnosed with CAD for the first time. Inclusion criteria were: (1) age ≥18 years; (2) presence of >30% stenosis in any major coronary artery on CTA; and (3) a diagnosis of stable angina, with guideline-directed treatment including antiplatelet and lipid-lowering therapy. Exclusion criteria included: (1) a prior diagnosis of CAD; (2) poor image quality preventing accurate CT-FFR and HRPC measurement; (3) cancer, heart failure, significant valvular disease, cardiomyopathy, primary arrhythmia, or other severe cardiac conditions; (4) non-cardiovascular causes of chest pain, such as pulmonary embolism or aortic dissection; and (5) stage 5 renal failure or patients undergoing dialysis. Informed consent was obtained from all patients, and the study was approved by the Ethics Committee of the First Hospital of Hebei Medical University (approval number: 20220362). The participant selection process is outlined in Figure 1, and a total of 222 patients were included. Baseline information, including sex, age, body mass index (BMI), medical history, and cardiovascular risk factors, was collected.

### CT-FFR and HRPC analysis

Coronary CTA images were obtained using either a Siemens SOMATOM Force dual-source CT scanner or a Toshiba Aquilion ONE 320-row CT scanner. Coronary CTA was performed using 320-row or dual-source CT scanners with prospective ECG-gated acquisition. Heart rate was controlled with oral or intravenous β-blockers when appropriate to maintain a stable rhythm below 60 bpm. Automatic tube-voltage selection (100–120 kVp) and tube-current modulation were applied according to body size. Iodinated contrast (300–370 mg I/mL, 50–80 mL) was injected at 4–6 mL/s followed by a 20 mL saline flush using bolus tracking (trigger threshold 120 HU in the ascending aorta). Images were reconstructed with 0.5–0.75 mm slice thickness using both standard and sharp kernels. CTA images were analyzed in a blinded manner by the CT team at the First Hospital of Hebei Medical University using Shukun v1.03 (Shukun Technology, Beijing, China), with quality control for segmentation accuracy and exclusion of scans with motion artifacts or inadequate contrast opacification.

The degree of coronary stenosis, CT-FFR values, and HRPC were assessed. Significant stenosis was defined as ≥50% narrowing of any major coronary artery. CT-FFR was calculated using a simplified machine-learning-enhanced algorithm. HRPC were identified as: (1) low-density plaque, defined as non-calcified regions with an attenuation <30 HU in three randomly selected areas within the plaque (23); (2) positive remodeling, defined by a remodeling index ≥1.1 (24); (3) napkin-ring sign, characterized by a low-attenuation core surrounded by a high-attenuation ring (25); and (4) spotty calcification, defined by an average attenuation >130 HU, a diameter <3 mm, a length <1.5× the vessel diameter, and a width <two-thirds of the vessel diameter (26).

### Data collection

Baseline demographic and clinical characteristics were recorded at hospital admission, including sex, age, BMI, smoking status, and alcohol use. Medical history included hypertension, diabetes, cerebrovascular disease, family history of cardiovascular disease, and hyperlipidemia, with detailed definitions provided in the appendix. Laboratory data were obtained from fasting blood samples taken at admission or within 24 h. Biomarkers included total cholesterol (TC), low-density lipoprotein cholesterol (LDL-C), high-density lipoprotein cholesterol (HDL-C), triglycerides (TG), apolipoprotein A1 (ApoA1), apolipoprotein B (ApoB), uric acid, creatinine, and high-sensitivity C-reactive protein (hs-CRP).

### Follow-up and outcome measurement

Patients were followed up at 1, 3, 6, and 12 months post-diagnosis, and annually thereafter, with follow-up ending on July 30, 2024. Data were collected through outpatient visits or telephone interviews with patients and their families, supplemented by electronic health records. To ensure data accuracy, follow-up data were cross-verified with electronic records. The primary outcome was the occurrence of major adverse cardiovascular events (MACE), which included all-cause death, acute myocardial infarction, unplanned revascularization, and rehospitalization for stable or unstable angina or heart failure.

### Statistical analysis

Baseline characteristics were compared across four groups categorized by CT-FFR and HRPC. Categorical variables were expressed as numbers and percentages, and continuous variables as mean ± standard deviation or median (interquartile range), depending on distribution. The Kolmogorov–Smirnov test was used to assess normality, and Levene's test to assess homogeneity of variances. Group comparisons for categorical variables were made using the chi-square test, while continuous variables were compared using one-way analysis of variance (ANOVA) or the Kruskal–Wallis test. The distribution of HRPC across CT-FFR groups was compared on a per-vessel basis to examine the relationship between CT-FFR values and HRPC. MACE risk was compared across groups on a per-patient basis. Kaplan–Meier survival curves were plotted to evaluate MACE incidence, and survival probabilities were compared using the log-rank test.

Multivariable Cox proportional hazards models were constructed to assess the independent predictive value of CT-FFR and HRPC for MACE risk. Covariates were selected according to clinical relevance and univariable Cox regression results with *P* < 0.30. The final multivariable Cox regression model included age, sex, smoking status, hyperlipidemia, coronary stenosis, and treatment modality, with CT-FFR and HRPC mutually adjusted for each other. Hazard ratios (HRs) with 95% confidence intervals (CIs) were reported. Subgroup analyses were performed to evaluate the consistency of associations across different patient subgroups, such as gender, diabetes, and hypertension.

The predictive performance and accuracy of CT-FFR and HRPC were evaluated by calculating the C-index (based on 10,000 bootstrap samples) and the area under the receiver operating characteristic (ROC) curve (AUC). Differences in performance between prediction models were compared using the C-index (bootstrap and *Z*-test comparison), AUC (DeLong test), net reclassification improvement (NRI), and integrated discrimination improvement (IDI). Statistical analyses were performed using R version 4.4.1 (R Foundation for Statistical Computing, Vienna, Austria). A *P*-value <0.05 was considered statistically significant.

## Results

### Baseline characteristics

A total of 222 patients were enrolled in the study, with a mean age of 65.1 ± 10.9 years; 78 (35.1%) were female. Fifty-one patients (23.0%) had diabetes, and 163 (73.4%) had hypertension. Based on their CT-FFR and HRPC values, the patients were divided into four groups: Group 1 (CT-FFR >0.8 and HRPC <2), Group 2 (CT-FFR >0.8 and HRPC ≥2), Group 3 (CT-FFR ≤0.8 and HRPC <2), and Group 4 (CT-FFR ≤0.8 and HRPC ≥2). The baseline characteristics of each group are shown in Table 1. In Group 4, the proportion of females was the lowest (24.5%, *P* = 0.002), serum creatinine levels were the highest (73.0 μmol/L, *P* = 0.029), and this group had the highest rates of PCI or CABG (46.4%, *P* = 0.001) and significant coronary stenosis (*P* < 0.001). There were no statistically significant differences between the groups in terms of age, hypertension, diabetes, BMI, smoking, alcohol consumption, or other biochemical indicators (all *P* > 0.05). Furthermore, the proportion of high-risk plaque characteristics—including low-density plaque, positive remodeling, and spotty calcification—was significantly higher in Group 4 compared to the other groups (*P* < 0.001).

**Table 1:** Clinical characteristics of individuals by CT-FFR and HRPC.

Variables	CT-FFR >0.8		CT-FFR ≤0.8		*P*
	HRPC <2	HRPC ≥2	HRPC <2	HRPC ≥2	
	Group 1 (*n* = 26)	Group 2 (*n* = 33)	Group 3 (*n* = 53)	Group 4 (*n* = 110)	
Age, years	65.7 (8.7)	63.8 (11.0)	64.5 (11.5)	65.7 (11.2)	0.796
Female, *n* (%)	15 (57.7)	11 (33.3)	25 (47.2)	27 (24.5)	0.002
Hypertension, *n* (%)	24 (92.3)	21 (63.6)	39 (73.6)	79 (71.8)	0.089
Diabetes, *n* (%)	7 (26.9)	5 (15.2)	15 (28.3)	24 (21.8)	0.512
Smoking, *n* (%)	21 (80.8)	21 (63.6)	40 (75.5)	65 (59.1)	0.069
Family history, *n* (%)	7 (26.9)	8 (24.2)	9 (17.0)	13 (11.8)	0.158
BMI, kg/m^2^	25.34 (3.87)	25.99 (2.66)	26.76 (3.53)	25.95 (3.30)	0.302
Hyperlipidemia, *n* (%)	12 (46.2)	17 (51.5)	16 (30.2)	38 (34.5)	0.156
TC, mmol/L	4.46 [3.62, 5.23]	5.00 [4.24, 5.71]	4.49 [3.84, 5.12]	4.82 [3.86, 5.46]	0.140
LDL-C, mmol/L	2.77 (0.80)	3.14 (0.76)	2.80 (0.77)	2.96 (0.84)	0.198
HDL-C, mmol/L	1.07 (0.20)	1.10 (0.22)	1.02 (0.22)	1.01 (0.23)	0.175
TG, mmol/L	1.33 [0.96, 2.06]	1.49 [1.10, 2.05]	1.32 [1.05, 1.70]	1.40 [0.98, 2.02]	0.851
ApoA1, g/L	1.25 (0.17)	1.26 (0.27)	1.24 (0.22)	1.23 (0.24)	0.959
ApoA1, g/L	0.76 (0.22)	0.85 (0.26)	0.76 (0.19)	0.83 (0.25)	0.139
Uric acid, µmol/L	317 (269, 369)	323 (271, 412)	349 (285, 379)	346 (291, 413)	0.410
Creatinine, μmol/L	61.3 [56.6, 71.0]	68.2 [57.1, 81.6]	72.0 [62.3, 80.9]	73.0 [64.2, 84.9]	0.029
Hs-CRP, mg/L	1.83 (1.68)	2.57 (4.69)	5.31 (16.05)	2.94 (4.77)	0.270
Treatment, *n* (%)					0.001
Medical therapy alone	23 (88.5)	28 (84.8)	40 (75.5)	59 (53.6)	
PCI	3 (11.5)	5 (15.2)	10 (18.9)	45 (40.9)	
CABG	0 (0.0)	0 (0.0)	3 (5.7)	6 (5.5)	
Significant stenosis^a^, *n* (%)					<0.001
0 vessel	11 (42.3)	9 (27.3)	8 (15.1)	8 (7.3)	
1 vessel	14 (53.8)	17 (51.5)	25 (47.2)	49 (44.5)	
2 vessels	1 (3.8)	6 (18.2)	14 (26.4)	41 (37.3)	
3 vessels	0 (0.0)	1 (3.0)	6 (11.3)	12 (10.9)	
≥1 vessels	15 (57.7)	24 (72.7)	46 (86.8)	103 (93.6)	<0.001
HRPC, *n* (%)					
Low attenuating plaque	1 (3.8)	13 (39.4)	5 (9.4)	45 (40.9)	<0.001
Positive remodeling	2 (7.7)	32 (97.0)	10 (18.9)	108 (98.2)	<0.001
Spotty calcification	0 (0.0)	15 (45.5)	1 (1.9)	64 (58.2)	<0.001
Napkin ring sign	1 (3.8)	26 (78.8)	2 (3.8)	107 (97.3)	<0.001

BMI, body mass index; TC, total cholesterol; LDL-C, low-density lipoprotein cholesterol; HDL-C, high-density lipoprotein cholesterol; TG, triglycerides; ApoA1, apolipoprotein A1; ApoB, apolipoprotein B; PCI, Percutaneous Coronary Intervention; CABG, Coronary Artery Bypass Grafting; HRPC, High-risk plaque characteristics; CT-FFR, CT-derived fractional ﬂow reserve.

a Significant stenosis: defined as ≥50% stenosis of a coronary artery as assessed by Coronary Computed Tomography Angiography.

### Comparison of plaque characteristics across FFR groups

On a per-patient basis, 163 (73.4%) of the 222 patients had at least one major artery with CT-FFR ≤0.8, and 165 (74.3%) exhibited at least one HRPC. Furthermore, 143 patients (64.4%) had two or more HRPC. Among the 666 coronary arteries analyzed, 290 (43.6%) had CT-FFR ≤0.8, and 247 (37.1%) exhibited at least one HRPC. Specifically, the number of vessels with each high-risk plaque feature was as follows: low-density plaque in 71 vessels (10.7%), napkin-ring sign in 180 vessels (27.0%), positive remodeling in 225 vessels (33.8%), and spotty calcification in 95 vessels (14.3%).

**Figure 2:**
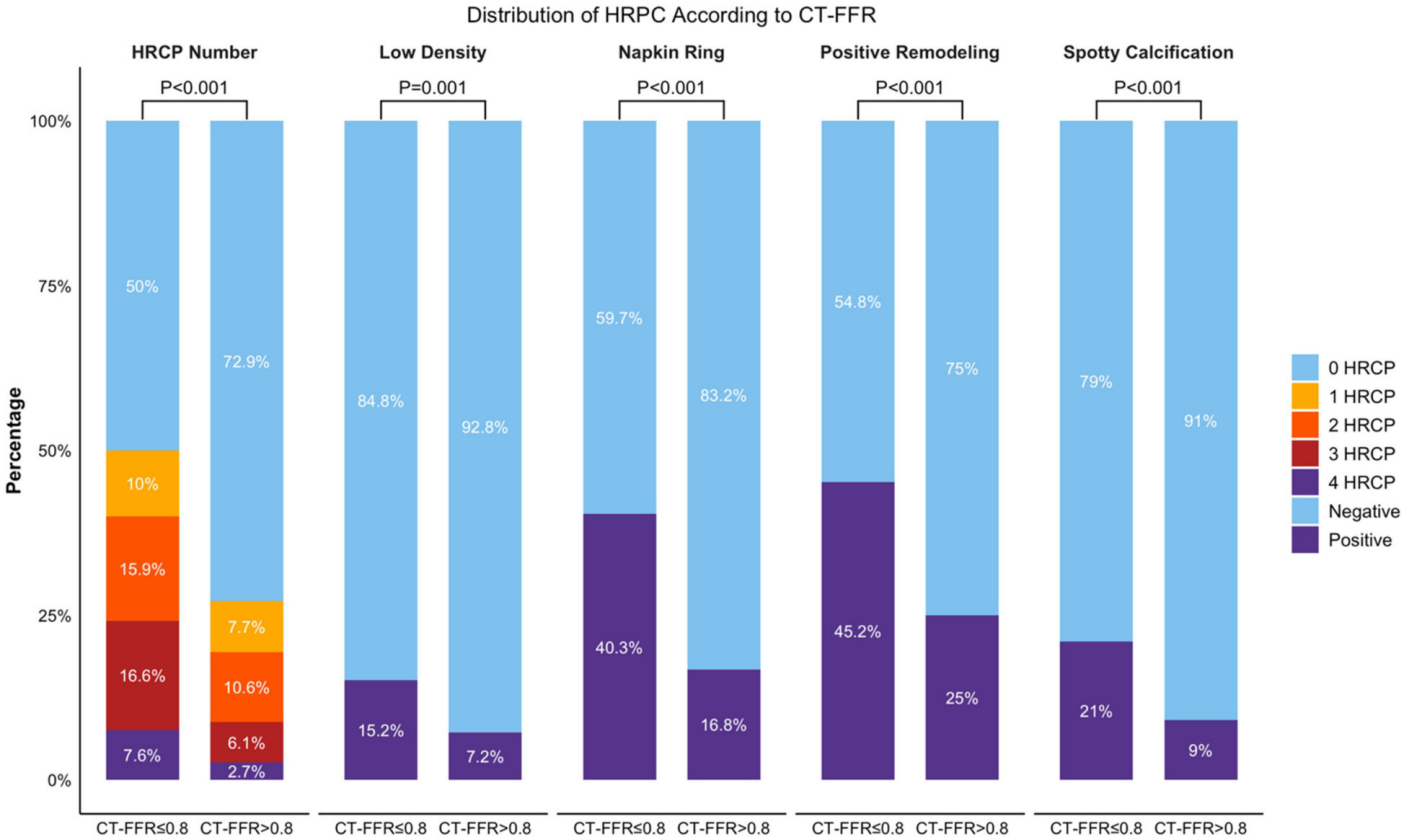
Distribution of HRPC counts and its characteristics in two CT-FFR groups. Bar graph comparing the total number of HRPC and the distribution of specific characteristics (Low Density, Napkin Ring, Positive Remodeling, Spotty Calcification) across CT-FFR groups (≤0.8 and >0.8). Percentages indicate the proportion of cases with 0–4 HRPCs. *P*-values show significant differences between groups for each characteristic. CT-FFR, CTA-derived fractional flow reserve; HRPC: high-risk plaque characteristics.

When comparing HRPC distribution across different CT-FFR groups, arteries with CT-FFR ≤0.8 had significantly more high-risk plaques, with 50% of these arteries containing HRPC, compared to 27.1% in arteries with CT-FFR >0.8 (*P* < 0.001). The proportion of arteries with 1–4 HRPC was 10%, 15.9%, 16.6%, and 7.6%, respectively, which was higher than the percentages observed in arteries with CT-FFR >0.8 (7.7%, 10.6%, 6.1%, and 2.7%; *P* < 0.001). Analyzing individual plaque characteristics revealed that arteries with CT-FFR ≤0.8 had significantly higher rates of low-density plaque, napkin-ring sign, positive remodeling, and spotty calcification compared to those with CT-FFR >0.8 (15.2% vs. 7.2%, 40.3% vs. 16.8%, 45.2% vs. 25%, and 21% vs. 9%, respectively; all *P* ≤ 0.001). These results are shown in Figure 2.

### Association of CT-FFR and HRPC with MACE incidence

**Figure 3:**
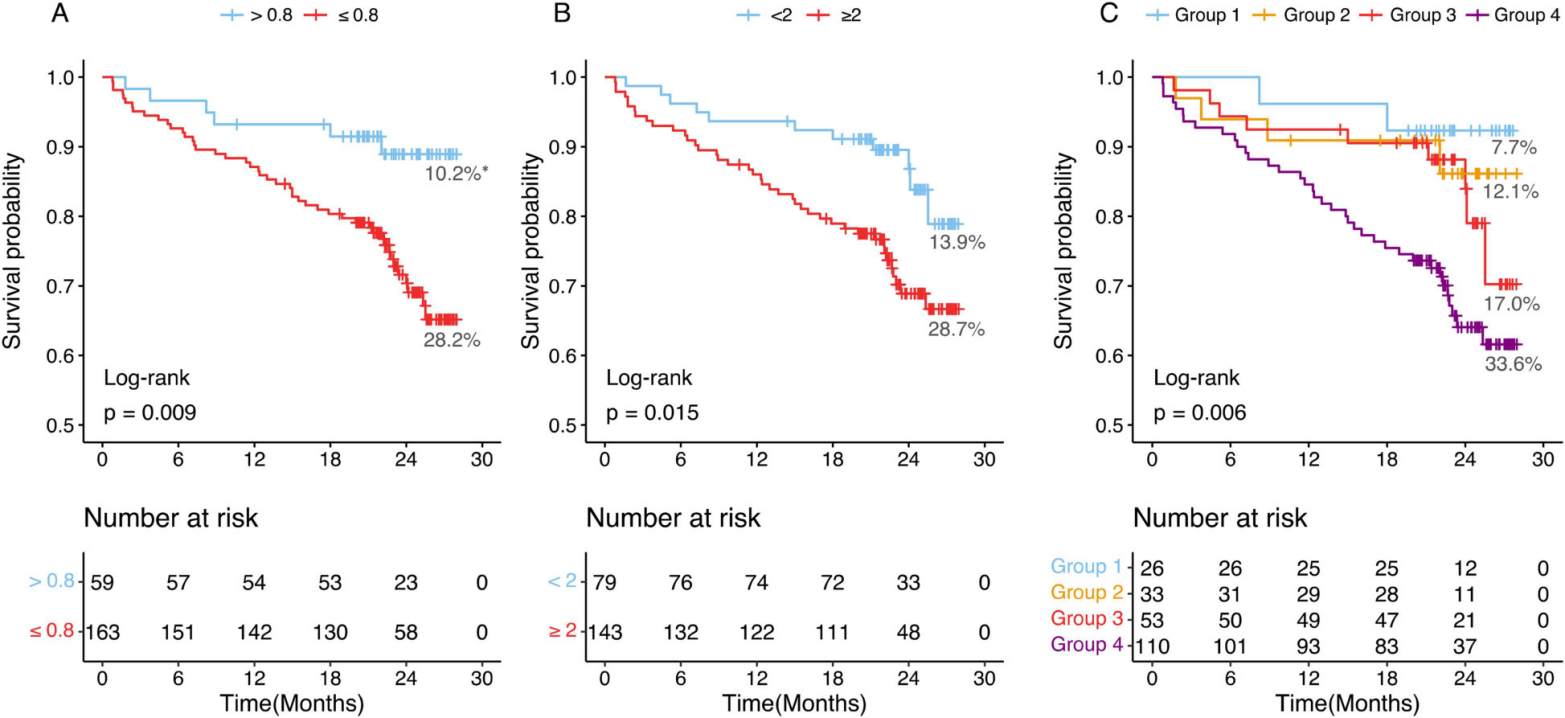
Kaplan–Meier curves and percentages of patients who experienced MACEs at different groups. **(A)** Patients with CT-FFR >0.8 or CT-FFR ≤0.8. **(B)** Patients with HRPC <2 or HRPC ≥2. **(C)** Patients were categorized into four groups based on CT-FFR and HRPC: Group 1 (CT-FFR >0.8 and HRPC <2), Group 2 (CT-FFR >0.8 and HRPC ≥2), Group 3 (CT-FFR ≤0.8 and HRPC < 2), and Group 4 (CT-FFR ≤0.8 and HRPC ≥2). CT-FFR, CTA-derived fractional flow reserve; HRPC, high-risk plaque characteristics. *Proportions of MACEs incidence in each group.

The median follow-up duration was 22.39 months (interquartile range: 20.26–25.11 months). During this period, 52 patients (23.4%) experienced major adverse cardiovascular events (MACE). Kaplan–Meier survival analysis (Figure 3) demonstrated that patients with CT-FFR ≤0.8 had a significantly higher MACE incidence (28.2%, 46 out of 163 patients) compared to those with CT-FFR >0.8 (10.2%, 6 out of 59 patients) (log-rank test, *P* = 0.009). Similarly, patients with HRPC ≥2 had a higher MACE incidence (28.7%, 41 out of 143 patients) compared to those with HRPC <2 (13.9%, 11 out of 79 patients) (*P* = 0.015). Combining both factors, patients in Group 4 (CT-FFR ≤0.8 and HRPC ≥2) exhibited the highest MACE incidence (33.6%, 37 out of 110 patients, *P* = 0.006), demonstrating a clear gradient of risk across different CT-FFR and HRPC combinations, as illustrated in Figure 3.

### Independent predictive value of CT-FFR and HRPC for MACE

Univariate Cox regression analysis results, presented in Appendix Table S1, revealed that both CT-FFR ≤0.8 and HRPC ≥2 were significantly associated with an increased risk of MACE. These findings were further supported by multivariate Cox regression analysis (Table 2), which adjusted for potential confounders such as age, sex, smoking status, hyperlipidemia, coronary stenosis, and treatment modality, as well as mutually adjusting for CT-FFR and HRPC. In this analysis, both CT-FFR ≤0.8 [hazard ratio [HR]: 2.62, 95% confidence interval (CI): 1.06–6.47, *P* = 0.036] and HRPC ≥2 (HR: 2.39, 95% CI: 1.20–4.77, *P* = 0.014) remained independent predictors of MACE. Moreover, combining both factors showed that patients in Group 4 had a 6.06-fold increased risk of MACE compared to those in Group 1 (95% CI: 1.38–26.52, *P* = 0.017), highlighting the combined prognostic value of these markers, as shown in Table 2.

**Table 2:** Association between CT-FFR, HRPC, and their combinations with the risk of MACE.

Groups	Model 1		Model 2		Model 3	
	HR (95% CI)	*P*	HR (95% CI)	*P*	HR (95% CI)	*P*
CT-FFR >0.8	Ref	—	Ref	—	Ref	—
CT-FFR ≤0.8	2.97 (1.27–6.95)	0.012	3.09 (1.31–7.28)	0.010	2.62 (1.06–6.47)^†^	0.036
HRPC <2	Ref	—	Ref	—	Ref	—
HRPC ≥2	2.24 (1.15–4.36)	0.017	2.46 (1.25–4.86)	0.009	2.39 (1.2–4.77)^§^	0.014
HRPC + CT-FFR						
Group 1	Ref	—	Ref	—	Ref	—
Group 2	1.77 (0.32–9.64)	0.512	2.01 (0.37–11.04)	0.421	2.27 (0.41–12.54)	0.349
Group 3	2.41 (0.52–11.13)	0.262	2.58 (0.56–11.96)	0.227	2.51 (0.53–11.89)	0.245
Group 4	5.09 (1.23–21.10)	0.025	6.03 (1.43–25.42)	0.014	6.06 (1.38–26.52)	0.017

HR, hazard ratio; CI, confidence interval; MACE, major adverse cardiovascular events; CT-FFR, computed tomography-derived fractional flow reserve; HRPC, high-risk plaque characteristics.

Group 1, CT-FFR >0.8&HRPC <2; Group 2, CT-FFR >0.8&HRPC ≥2; Group 3, CT-FFR ≤0.8&HRPC <2; Group 4, CT-FFR ≤0.8&HRPC ≥2.

Model 1: Unadjusted.

Model 2: Model 1 + age, gender and smoking.

Model 3: Model 2 + hyperlipidemia, significant stenosis, revascularization (percutaneous coronary intervention or coronary artery bypass grafting), and additionally adjusted for HRPC and CT-FFR where applicable.

† Additional adjusted for HRPC (<2 or ≥2).

§ Additional adjusted for CT-FFR (>0.8 or ≤0.8).

**Figure 4:**
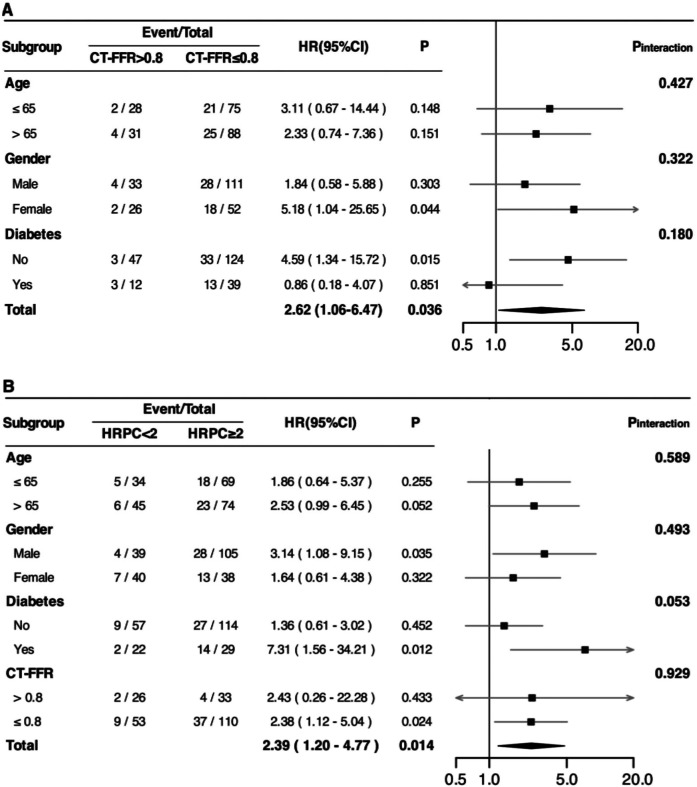
Associations between CT-FFR **(A)** and HRPC **(B)** and the risk of experiencing MACE among patients with newly diagnosed CCS in different subgroups. HRs were adjusted for age, gender, smoking status, hyperlipidemia, significant coronary stenosis, and revascularization (percutaneous coronary intervention or coronary artery bypass grafting). Additionally, adjustments were made for HRPC (in **A**) and CT-FFR (in **B**) where applicable. HR, hazard ratio; CI, confidence interval; MACE, major adverse cardiovascular events; CCS, chronic coronary syndrome; CT-FFR, computed tomography-derived fractional flow reserve; HRPC, high-risk plaque characteristics;.

Subgroup analyses (Figure 4) were conducted to explore potential variations in the predictive value of CT-FFR and HRPC across different patient groups. However, no significant interaction effects were found for either CT-FFR or HRPC (all *P* for interaction >0.05). Additionally, HRPC's predictive value remained consistent across different CT-FFR strata (*P* for interaction = 0.929), confirming the robustness of these markers in predicting MACE.

### Improvement of cardiovascular risk prediction

Incorporating CT-FFR and HRPC into traditional cardiovascular risk prediction models significantly enhanced their predictive performance (Table 3). The base model, which included age, sex, smoking status, hyperlipidemia, significant coronary stenosis, and revascularization, had a C-index of 0.621 and an area under the receiver operating characteristic curve (AUC) of 0.641. Adding either CT-FFR or HRPC individually improved model performance, with significant improvements in both the net reclassification improvement (NRI) and integrated discrimination improvement (IDI) (all NRI *P*-values ≤0.006; all IDI *P*-values ≤0.012).

**Table 3:** Improvement in MACE risk reclassiﬁcation and discrimination with CT-FFR and HRPC.

Reference	Comparison model	C index (95%CI)^§^	*P*	AUC	*P*	NRI (95% CI)	*P*	IDI (95% CI)	*P*
BM^†^	—	0.621 (0.533–0.709)^‡^	—	0.641^‡^	—	—	—	—	—
BM	BM + CT-FFR	0.653 (0.574–0.731)	0.157	0.681	0.143	0.393 (0.170–0.615)	**<0.001**	0.026 (0.006–0.047)	**0.012**
BM	BM + HRPC	0.664 (0.583–0.746)	0.078	0.677	0.201	0.377 (0.111–0.643)	**0.006**	0.030 (0.008–0.052)	**0.007**
BM	BM + CT-FFR + HRPC	0.688 (0.613–0.762)	**0.021**	0.712	**0.038**	0.532 (0.248–0.816)	**<0.001**	0.056 (0.025–0.087)	**<0.001**
BM + CT-FFR	BM + CT-FFR + HRPC	—	0.237	—	0.180	0.377 (0.111–0.643)	**0.006**	0.030 (0.007–0.053)	**0.010**
BM + HRPC	BM + CT-FFR + HRPC	—	0.214	—	0.110	0.393 (0.170–0.615)	**<0.001**	0.026 (0.005–0.047)	**0.017**

HR, hazard ratio; CI, confidence interval; MACE, Major adverse cardiovascular events; CT-FFR, Computed tomography-derived fractional flow reserve; HRPC, High-risk plaque characteristics; C-index, Harrell's concordance index; AUC, area under the receiver operating characteristic curve; NRI, net reclassification improvement; IDI, integrated discrimination improvement.

Bold text indicates that the result is statistically significant.

† BM (The basic model) included age, gender, smoked or smoking, hyperlipidemia, one or more vessels with stenosis≥50% and revascularization (percutaneous coronary intervention or coronary artery bypass grafting).

§ C-index calculated using 10,000 bootstrap samples.

‡ C-index and AUC values in the first row correspond to the Basic Model (BM). In subsequent rows, these values correspond to the Comparison Models.

As shown in Table 3, the addition of CT-FFR and HRPC to the base model significantly improved model performance. Relative to the base model (BM), the following incremental changes were observed: BM → BM + CT-FFR: ΔC-index: +0.032, ΔAUC: +0.040, NRI: 0.393 (*P* < 0.001), IDI: 0.026 (*P* = 0.012); BM → BM + HRPC: ΔC-index: +0.043, ΔAUC: +0.036, NRI: 0.377 (*P* = 0.006), IDI: 0.030 (*P* = 0.007); BM → BM + CT-FFR + HRPC: ΔC-index: +0.067, ΔAUC: +0.071, NRI: 0.532 (*P* < 0.001), IDI: 0.056 (*P* < 0.001). Compared with either predictor alone, the combined model demonstrated superior reclassification (NRI: 0.377–0.393) and discrimination (IDI: 0.026–0.030). All C-index values were obtained from 10,000 bootstrap samples, and continuous NRI and IDI were used.

**Figure 5:**
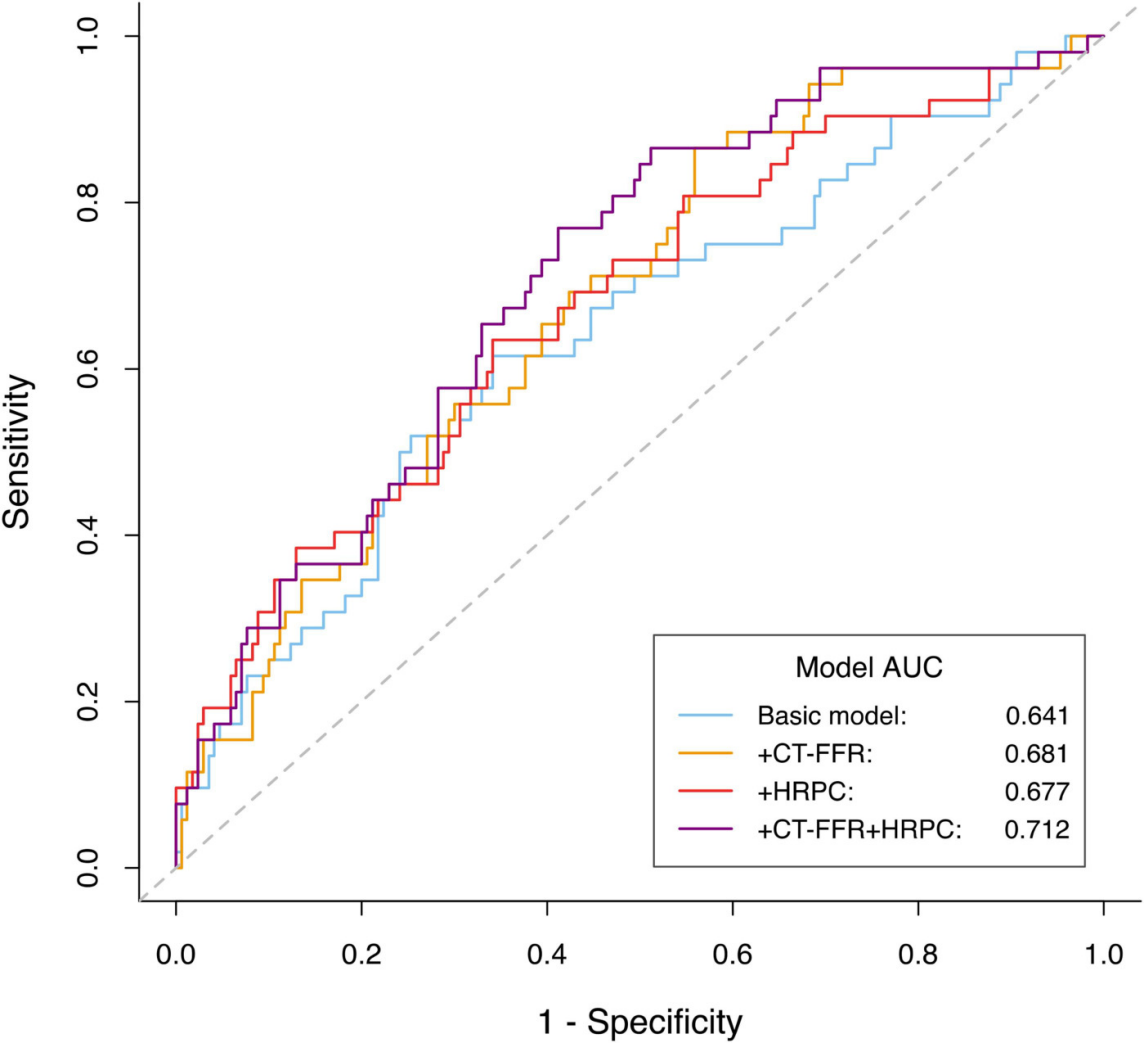
ROC curves comparing the predictive performance of different models for predicting MACE. The basic model included age, gender, smoked or smoking, hyperlipidemia, one or more vessels with stenosis≥50% and revascularization (percutaneous coronary intervention or coronary artery bypass grafting). MACE, major adverse cardiovascular events; CT-FFR, computed tomography-derived fractional flow reserve; HRPC, high-risk plaque characteristics.

Notably, the greatest improvement was observed when both CT-FFR and HRPC were added simultaneously. Compared to adding either marker alone, the combination resulted in significantly greater improvements in NRI and IDI (all *P*-values ≤0.006 for NRI; all *P*-values ≤0.017 for IDI). Furthermore, adding both CT-FFR and HRPC led to significant improvements over the base model in both C-index, increasing from 0.621 to 0.688 (*P* = 0.021), and AUC, increasing from 0.641 to 0.712 (*P* = 0.038). These results highlight the superior predictive power of using both CT-FFR and HRPC together to identify high-risk patients and enhance cardiovascular risk prediction (Table 3, Figure 5).

## Discussion

To our knowledge, this is the first study to evaluate the combined prognostic value of AI-based CT-FFR and HRPC for risk stratification in patients with CAD. Our findings highlight three key points: First, vessels with CT-FFR ≤0.8 were significantly more likely to exhibit HRPC. Second, both CT-FFR ≤0.8 and HRPC ≥2 were independent predictors of MACE, even after adjusting for multiple confounding factors. Third, patients with both CT-FFR ≤0.8 and HRPC ≥2 had the worst prognosis, with the combination of these two metrics providing the highest predictive accuracy. These results suggest that the simultaneous use of CT-FFR and HRPC, both of which can be easily obtained from coronary CTA, may offer significant clinical utility for risk stratification.

The overall MACE incidence in this study (23.4% over a median follow-up of 22 months) was higher than that reported in large multicenter trials such as DISCHARGE and SCOT-HEART, mainly due to differences in endpoint definitions and study populations. Our definition of MACE was broader, including cardiovascular death, nonfatal myocardial infarction, revascularization (PCI or CABG), and rehospitalization for heart failure or severe angina, whereas those prior trials focused primarily on hard endpoints such as all-cause death, cardiovascular death, and nonfatal stroke. Moreover, our study enrolled hospitalized patients with chronic coronary syndrome (CCS) who generally present with more advanced disease, multiple comorbidities, and higher intervention rates than the outpatient or lower-risk populations in DISCHARGE and SCOT-HEART, which reasonably explains the higher cumulative event rate observed during a comparable follow-up period.

Our findings demonstrated a clear association between CT-FFR and HRPC. Vessels with CT-FFR ≤0.8 were significantly more likely to have HRPC, and the proportion of vessels with multiple HRPC was higher in this group. Each high-risk plaque feature—including low-density plaque, napkin-ring sign, positive remodeling, and spotty calcification—was more frequent in vessels with CT-FFR ≤0.8. These observations are consistent with prior research, including the PACIFIC trial (27), which demonstrated a similar association with invasive FFR. Other studies, including those by Otaki et al. (28) and Lee et al. (29), confirmed that HRPC are associated with both CT-based and invasive FFR, regardless of stenosis severity. Most prior studies relied on 64-slice or higher CT scanners (7, 29–31), whereas our study used 320-slice or dual-source CT scanners, which may have improved the detection of high-risk plaques (32–34).

CT-FFR has been widely validated as an independent predictor of adverse outcomes in patients with stable CAD. It offers a functional assessment of ischemia, complementing anatomical measures of stenosis severity. Our findings are consistent with previous studies showing that patients with CT-FFR ≤0.8 have worse outcomes compared to those with higher values (14, 35, 36). Importantly, AI-based CT-FFR is a non-invasive tool that provides quick and reliable results, reducing unnecessary invasive testing (37, 38), lowering healthcare costs, and offering valuable prognostic insights (39, 40). Previous research has demonstrated that AI-based CT-FFR performs well in identifying functional ischemia, further supporting its clinical utility (41, 42). By incorporating CT-FFR into routine clinical practice, it may be possible to avoid up to 57% of unnecessary invasive procedures (38), making it especially beneficial in high-risk populations or in situations where invasive testing poses additional risk (43, 44).

Coronary artery stenosis and ischemia alone do not fully capture cardiovascular risk, as they overlook key plaque characteristics crucial for predicting outcomes (16, 45–47). For example, while only 42% of patients with acute myocardial infarction had >50% stenosis, 92% showed evidence of coronary plaque (48). Beyond ischemia, HRPC identified via CTA have proven essential for diagnosing CAD and predicting outcomes, consistent with prior studies (19, 30, 49). The ROMICAT-II trial found that HRPC independently predicted acute myocardial infarction, even in patients with normal ECG and troponin levels (30). Long-term studies with follow-up periods of 7.8–10.5 years have also demonstrated that spotty calcification, low-density plaque, and napkin-ring sign strongly predict MACE, beyond stenosis and traditional risk factors (6, 7, 50). In our study, we used HRPC ≥2 as a threshold for comprehensive risk assessment, a method supported by a meta-analysis of 30 studies that demonstrated its highest predictive accuracy for MACE (19). Additionally, HRPC ≥2 was more effective for risk stratification than calcium scores or the Framingham risk score in patients with acute stroke and no prior CAD history (20).

Crucially, our findings demonstrate that CT-FFR and HRPC are not only correlated but also provide complementary information for predicting MACE in CAD patients. Incorporating either metric into traditional risk models enhanced predictive accuracy, with the most significant improvement observed when both were combined. The addition of both CT-FFR and HRPC significantly increased the C-index, AUC, NRI, and IDI (all *P* ≤ 0.038), whereas neither marker alone had such a profound effect. This suggests that ischemic burden and plaque vulnerability are independent yet synergistic risk factors for adverse outcomes in CAD (10, 51). For instance, even in patients with FFR >0.8, the presence of HRPC increased the risk of MACE (11). Additionally, HRPC adds prognostic value in patients with FFR <0.8 before PCI and FFR >0.8 after PCI, with HRPC ≥2 predicting worse outcomes (52). The SCOT-HEART trial similarly demonstrated that patients with both significant stenosis and high-risk plaques were at the greatest risk (53), a pattern also seen in post-myocardial infarction patients (10) and those with multivessel disease (49). Furthermore, the NXT sub-study confirmed that CT-FFR maintains high diagnostic accuracy for ischemic lesions across various levels of plaque calcification, reinforcing its utility in diagnosing functionally significant ischemia (54).

In this study, the combination of CT-FFR and HRPC provided incremental prognostic value beyond either parameter alone. CT-FFR reflects the functional significance of coronary stenosis, whereas HRPC captures plaque vulnerability; integrating both enables a more comprehensive assessment of ischemic and morphological risk. The complementary prognostic information from CT-FFR and HRPC is supported by prior landmark work: noninvasive CT-FFR has shown strong diagnostic performance (42), while CTA-defined high-risk plaque features—including positive remodeling, low-attenuation plaque, and spotty calcification—predict future acute coronary syndromes (55); the napkin-ring sign further indicates advanced atherosclerotic lesions on histology (25). Reviews underscore that combining functional and morphological CT metrics can enhance prognostic accuracy and decision-making (54). Outcome-oriented studies of FFRCT-guided strategies also support clinical utility in real-world settings (40). Together, these data and our findings suggest that integrated CT-FFR plus HRPC evaluation may better identify high-risk CCS inpatients who could benefit from intensified therapy or closer surveillance, supporting individualized inpatient management.

These findings have significant clinical implications. Combining CT-FFR with HRPC offers a more comprehensive risk assessment, particularly in patients with CT-FFR ≤0.8 and HRPC ≥2, where the combined approach significantly enhances risk stratification. This method can help guide clinical decision-making and optimize treatment plans. Additionally, both CT-FFR and HRPC are non-invasive and can be derived from the same coronary CTA images, making this approach both cost-effective and practical, especially in resource-limited settings. Although our study focused on a subset of newly diagnosed, non-emergency CAD patients, excluding those with myocardial infarction, the integration of these two metrics may have broader implications for risk prediction across the full spectrum of CAD. This combined approach holds potential for improving risk assessment and treatment strategies in a wider range of CAD populations. Nevertheless, larger studies are required to further validate these findings and explore their full clinical potential.

### Limitations

This study has several limitations. First, being a single-center study with a relatively small sample size, its findings may not be broadly generalizable. Second, as this study focused primarily on CT-FFR and HRPC, and only a portion of patients underwent invasive coronary angiography (ICA), we did not include ICA results in our analysis. However, we did adjust for coronary stenosis based on CTA findings. As a registry-based observational study, no prospective sample-size estimation was performed. The limited number of MACE events may have reduced the statistical power, particularly for subgroup and interaction analyses; therefore, these results should be interpreted as exploratory. Lastly, as an observational study, this research does not explore the effects of therapeutic interventions in patients with high HRPC and low CT-FFR. Future studies should focus on larger multicenter cohorts and interventional research to validate the prognostic utility of combining HRPC and CT-FFR.

## Conclusion

The combination of AI-derived CT-FFR and HRPC significantly improves cardiovascular risk prediction in newly diagnosed chronic coronary syndrome. Patients with both CT-FFR ≤0.8 and HRPC ≥2 are at the highest risk of adverse outcomes, and combining these metrics enhances risk stratification and supports more informed clinical decisions. While these findings are promising, larger multicenter studies are needed to confirm these results and further establish the clinical value of integrating CT-FFR and HRPC.

## Data Availability

The original contributions presented in the study are included in the article/Supplementary Material, further inquiries can be directed to the corresponding authors.
