# Supplementary material for: Combined prognostic value of AI-derived CT-FFR and high-risk plaque characteristics in patients with newly diagnosed chronic coronary syndrome: a prospective cohort study
Source: Front Cardiovasc Med. 2025 Dec 3;12:1674126. doi: 10.3389/fcvm.2025.1674126 (PMC12708924; doi:10.3389/fcvm.2025.1674126)
Supplement: Supplementary file 1 [file Datasheet1.pdf]

I Definitions

In this study, all patients were diagnosed with coronary artery disease (CAD), based on the diagnostic criteria established by the American College of Cardiology (ACC) /the American Heart Association (AHA). Specifically, the criteria require confirmation of at least one coronary artery stenosis exceeding 30% through coronary computed tomography angiography (CTA). Initial diagnosis of CAD refers to cases previously unconfirmed by coronary angiography or CTA. Furthermore, all patients included in the study exhibited symptoms of angina with varying degrees of severity upon admission.

BMI was calculated as bodyweight in kilograms divided by the square of the body height in meters (kg/m^2). Smoker was defined as smoking at least one cigarette per day at the time of the survey or before. Alcohol consumption was defined as anyone who consumed alcohol once a week or more. Hypertension was defined as an average of two measurements of systolic blood pressure ≥140 mmHg or diastolic blood pressure ≥90 mmHg, or current use of antihypertensive agents. Diabetes was defined as FPG ≥7.0 mmol/L, or current use of insulin or oral antidiabetic agents, or the participants reported history of diabetes.

Sensitivity Analysis for Cardiac-Specific Mortality

We performed an additional sensitivity analysis by excluding the single participant with an indeterminate cause of death and redefining mortality as cardiac-specific death instead of all-cause mortality. Although this analysis was not part of the original study protocol and is therefore not included in the main text, we provide the results here for completeness. The survival patterns and

risk stratification across CT-FFR and HRPC groups were highly consistent with the primary analysis, supporting the robustness of our findings. The corresponding tables (Table S3 and Table S4) and figure (Figure S2) summarize the results of this cardiac-specific MACE analysis.

II Supplemental tables

Table S1 Hazard ratio of the unadjusted factor for MACE risk

	Risk of MACE		
	**HR (95% CI)**	**β**	**p**
Age, years	1.011 (0.985-1.038)	0.011	0.407
Female, n(%)	1.195 (0.684-2.091)	0.179	0.531
Hypertension, n(%)	1.328 (0.683-2.584)	0.284	0.403
Diabetes, n(%)	1.642 (0.911-2.96)	0.496	0.099
Smoking, n(%)	1.382 (0.758-2.520)	0.324	0.291
Family history, n(%)	1.297 (0.666-2.523)	0.260	0.444
BMI, kg/m^2	1.024 (0.943-1.111)	0.023	0.576
Hyperlipidemia, n(%)	1.394 (0.806-2.410)	0.332	0.235
TC, mmol/L	1.024 (0.818-1.281)	0.023	0.839
LDL-C, mmol/L	1.026 (0.734-1.432)	0.025	0.882
HDL-C, mmol/L	0.776 (0.226-2.662)	-0.253	0.687
TG, mmol/L	1.049 (0.867-1.270)	0.048	0.622
ApoA1, g/L	0.976 (0.298-3.201)	-0.024	0.968
ApoB, g/L	1.111 (0.361-3.423)	0.106	0.854
Uric acid, µmol/L	1.001 (0.998-1.004)	0.001	0.534
Hs-CRP, mg/L	0.995 (0.960-1.031)	-0.005	0.768
Creatinine, µmol/L	1.005 (0.995-1.015)	0.005	0.334
Treatment, n (%)			
Medical therapy alone	reference	-	-
PCI	1.393 (0.768-2.525)	0.331	0.275
CABG	2.886 (1.120-7.441)	1.060	0.028
No. of vessels with stenosis≥50%, n (%)			
0	reference	-	-
1	1.138 (0.457-2.833)	0.129	0.782
2	1.685 (0.668-4.248)	0.522	0.269
3	2.958 (1.026-8.528)	1.084	0.045
≥1	2.207 (0.796-6.121)	0.792	0.128
No. of vessels with stenosis≥70%, n (%)	1.409 (0.796-2.495)	0.343	0.240
0	reference	-	-
1	1.406 (0.778-2.542)	0.341	0.259
2	1.232 (0.457-3.322)	0.209	0.680

3	5.861 (0.777-44.23)	1.768	0.086
≥1	1.409 (0.796-2.495)	0.343	0.240
HRPC, n (%)			
Low attenuating plaque	1.239 (0.693-2.212)	0.214	0.470
Positive remodeling	2.384 (1.162-4.890)	0.869	0.018
Spotty calcification	1.915 (1.112-3.298)	0.650	0.019
Napkin ring sign	2.063 (1.101-3.865)	0.724	0.024
No. of HRPC			
0	reference	-	-
1	0.559 (0.121-2.588)	-0.582	0.457
2	1.428 (0.592-3.446)	0.356	0.428
3	2.187 (0.989-4.834)	0.782	0.053
4	2.439 (1.010-5.889)	0.892	0.047
No. of CT-FFR≤0.8			
0	reference	-	-
1	2.300 (0.907-5.834)	0.833	0.079
2	3.362 (1.304-8.667)	1.213	0.012
3	3.837 (1.474-9.986)	1.345	0.006

BMI, body mass index; TC, total cholesterol; LDL-C, low-density lipoprotein cholesterol; HDL-C, high-density lipoprotein cholesterol; TG, triglycerides; ApoA1, apolipoprotein A1; ApoB, apolipoprotein B;PCI, Percutaneous Coronary Intervention; CABG, Coronary Artery Bypass Grafting; HRPC, High-risk plaque characteristics; CT-FFR, CT-derived fractional flow reserve.

Table S2 Distribution of MACE components

	CT-FFR > 0.8		CT-FFR ≤ 0.8	
	HRPC < 2	HRPC ≥ 2	HRPC < 2	HRPC ≥ 2
Events	**Group 1 (n=26)**	**Group 2 (n=33)**	**Group 3 (n=53)**	**Group 4 (n=110)**
All-cause death	0	1	0	3
Non-fatal MI	0	1	0	7
Unplanned revascularization	0	1	3	9
Rehospitalization for angina or heart failure	2	1	6	18

Table S3 Association between CT-FFR, HRPC, and their combinations with the risk of MACE (Cardiovascular death, MI, reperfusion，angina and heart failure)

	Model1		Model2		Model3	
	**HR (95% CI)**	***P***	**HR (95% CI)**	***P***	**HR (95% CI)**	***P***
CT-FFR>0.8	Ref	-	Ref	-	Ref	-
CT-FFR≤0.8	3.56 (1.42-8.97)	0.007	3.76 (1.48-9.51)	0.005	3.3 (1.25-8.69)[†]	0.016
HRPC<2	Ref	-	Ref	-	Ref	-
HRPC≥2	2.19 (1.12-4.27)	0.021	2.42 (1.22-4.78)	0.011	2.28 (1.14 -4.57)[§]	0.02
HRPC+CT-FFR						
Group 1	Ref	-	Ref	-	Ref	-
Group 2	1.33 (0.22 - 7.94)	0.757	1.62 (0.27-9.82)	0.598	1.62 (0.27-9.82)	0.598
Group 3	2.41 (0.52-11.15)	0.261	2.54 (0.54-12)	0.238	2.54 (0.54-12)	0.238
Group 4	5.09 (1.23-21.13)	0.025	6.13 (1.4-26.75)	0.016	6.13 (1.4-26.75)	0.016

HR, hazard ratio; CI, confidence interval; MACE, Major adverse cardiovascular events; CT-FFR, Computed tomography-derived fractional flow reserve; HRPC, High-risk plaque characteristics.

Group 1, CT-FFR>0.8&HRPC<2; Group 2, CT-FFR>0.8&HRPC≥2; Group 3, CT-FFR≤0.8&HRPC<2; Group 4, CT-FFR≤0.8&HRPC≥2.

Model 1: Unadjusted.

Model 2: Model 1 + age, gender and smoking.

Model 3: Model 2 + hyperlipidemia, significant stenosis, revascularization (percutaneous coronary intervention or coronary artery bypass grafting), and additionally adjusted for HRPC and CT-FFR where applicable.

[†]Additional adjusted for HRPC (<2 or ≥2).

[§]Additional adjusted for CT-FFR (>0.8 or ≤0.8).

Table S4 Improvement in MACE (Cardiovascular death, MI, reperfusion，angina and heart failure) risk reclassification and discrimination with CT-FFR and HRPC

Reference	Comparison model	C index (95%CI)[§]	P	AUC	P	NRI (95% CI)	P	IDI (95% CI)	P
BM[†]	-	0.613(0.525-0.701)[‡]	-	0.630[‡]	-	-	-	-	-
BM	BM+CT-FFR	0.652(0.572-0.731)	0.123	0.684	0.073	0.393(0.170-0.615)	**<0.001**	0.031(0.008-0.054)	**0.008**
BM	BM+HRPC	0.652(0.571-0.733)	0.122	0.666	0.238	0.377(0.111-0.643)	**0.006**	0.029(0.008-0.050)	**0.007**
BM	BM+CT-FFR+HRPC	0.684(0.610-0.758)	**0.020**	0.711	**0.031**	0.532(0.248-0.816)	**<0.001**	0.060(0.027-0.092)	**<0.001**
BM+CT-FFR	BM+CT-FFR+HRPC	-	0.115	-	0.180	0.377(0.111-0.643)	**0.006**	0.029(0.007-0.051)	**0.010**
BM+HRPC	BM+CT-FFR+HRPC	-	0.129	-	0.110	0.393(0.170-0.615)	**<0.001**	0.031(0.006-0.055)	**0.013**

HR, hazard ratio; CI, confidence interval; MACE, Major adverse cardiovascular events; CT-FFR, Computed tomography-derived fractional flow reserve; HRPC, High-risk plaque characteristics; C-index, Harrell's concordance index; AUC, Area Under the Receiver Operating Characteristic Curve; NRI, net reclassification improvement; IDI, integrated discrimination improvement.

[†]BM (The basic model) included age, gender, smoked or smoking, hyperlipidemia, one or more vessels with stenosis≥50% and revascularization (percutaneous coronary intervention or coronary artery bypass grafting).

[§]C-index calculated using 10,000 bootstrap samples.

‡ C-index and AUC values in the first row correspond to the Basic Model (BM). In subsequent rows, these values correspond to the Comparison Models.

II Supplemental figures

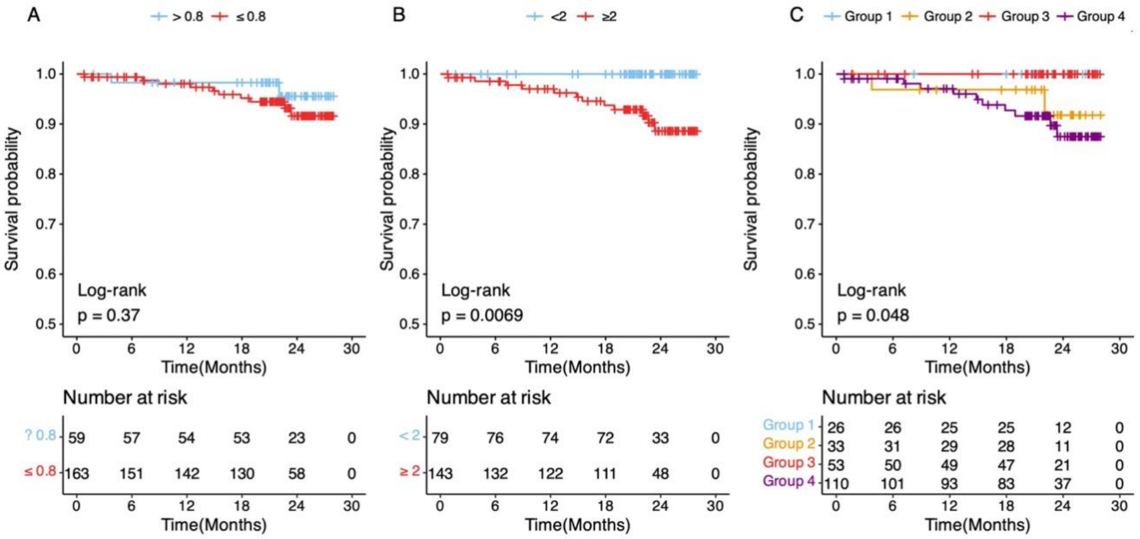

Figure S1 Kaplan–Meier curves and percentages of patients who experienced **hard cardiac events** (death and nonfatal MI) at different Groups. A: Patients with CT-FFR > 0.8 or CT-FFR ≤ 0.8. B: Patients with HRPC < 2 or HRPC ≥ 2. C: Patients were categorized into four groups based on CT-FFR and HRPC: Group 1 (CT-FFR > 0.8 and HRPC < 2), Group 2 (CT-FFR > 0.8 and HRPC ≥ 2), Group 3 (CT-FFR ≤ 0.8 and HRPC < 2), and Group 4 (CT-FFR ≤ 0.8 and HRPC ≥ 2). CT-FFR, CTA-derived fractional flow reserve; HRPC: high-risk plaque characteristics.

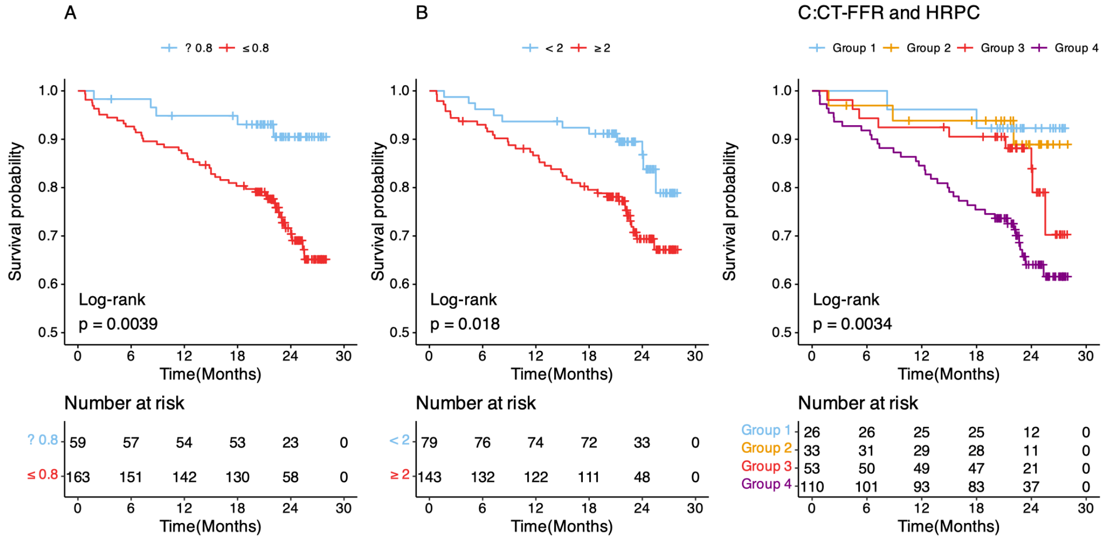

Figure S2 Kaplan–Meier curves and percentages of patients who experienced MACE (Cardiovascular death, MI, reperfusion, angina and heart failure) at different Groups. A: Patients with CT-FFR > 0.8 or CT-FFR ≤ 0.8. B: Patients with HRPC < 2 or HRPC ≥ 2. C: Patients were categorized into four groups based on CT-FFR and HRPC: Group 1 (CT-FFR > 0.8 and HRPC < 2), Group 2 (CT-FFR > 0.8 and HRPC ≥ 2), Group 3 (CT-FFR ≤ 0.8 and HRPC < 2), and Group 4 (CT-FFR ≤ 0.8 and HRPC ≥ 2). CT-FFR, CTA-derived fractional flow reserve; HRPC: high-risk plaque characteristics.
